# PerAF-based resting-state fMRI classifier for minimal hepatic encephalopathy

**DOI:** 10.3389/fneur.2025.1603396

**Published:** 2025-08-26

**Authors:** Yunli Zhang, Zhai Huang, Bin Qin, Shiting Tang, Peng Huang, Xiaogang Tang, Xuezhen Lin, Yayuan Liu, Xuhui Deng, Feiqi Zhu, Shihui Xing, Zhijian Liang

**Affiliations:** ^1^Department of Intensive Care Unit, Guangxi Academy of Medical Sciences, The People's Hospital of Guangxi Zhuang Autonomous Region, Nanning, China; ^2^Department of Neurology, The First Affiliated Hospital of Guangxi Medical University, Nanning, China; ^3^Department of Neurology, Liuzhou People's Hospital, Liuzhou, China; ^4^Department of Neurology, Second Affiliated Hospital of Guangxi Medical University, Nanning, China; ^5^Department of Neurology, Minzu Hospital of Guangxi Zhuang Autonomous Region, Nanning, China; ^6^Department of Neurology, Yuebei People's Hospital, Guangdong, China; ^7^Department of Neurology, Luohu Hospital of Shenzhen, Guangdong, China; ^8^Department of Neurology, The First Affiliated Hospital, Sun Yat-sen University, Guangdong, China

**Keywords:** minimal hepatic encephalopathy, resting-state fMRI, baseline regional brain activity, percent amplitude of fluctuation, machine learning

## Abstract

**Background:**

Minimal hepatic encephalopathy (MHE) is a common cognitive impairment in patients with end-stage liver cirrhosis. However, the selection of sensitive biomarkers and the establishment of reliable diagnostic methods are currently challenging. We aimed to explore the abnormal spontaneous brain activity in patients with MHE and evaluate the clinical diagnostic value of four indicators for MHE using the support vector machine (SVM) method.

**Methods:**

A total of 45 MHE patients and 40 healthy controls were enrolled. Amplitude of low frequency fluctuation (ALFF), fractional amplitude of low frequency fluctuation (fALFF), percentage amplitude of low frequency fluctuation (PerAF), and regional homogeneity (ReHo) were used to evaluate local spontaneous brain activity. SVM analysis was used to construct the classification model and evaluate the diagnostic value.

**Results:**

Two-sample *t*-test and SVM analysis showed that, compared with the healthy control group, MHE patients had decreased ALFF values in the left angular gyrus, right inferior temporal gyrus, left postcentral gyrus, precentral gyrus, and right supplementary motor area. These regions indicated moderate classification efficacy (AUC = 0.75). Decreased ReHo metrics in the right anterior cingulate and paracingulate gyri also showed general discriminative power (AUC = 0.72). fALFF metrics, whether analyzed independently or combined with other indicators, exhibited limited classification performance (AUC < 0.70). Decreased PerAF metrics in the right superior parietal lobule, right dorsolateral prefrontal cortex, and right middle frontal gyrus achieved a good classification accuracy rate (AUC value 0.83; accuracy 81.18%; sensitivity 75.56%; specificity 87.50%), outperforming other functional metrics.

**Conclusion:**

We found that decreased mean PerAF in the right supramarginal gyrus, right dorsolateral superior frontal gyrus, and right middle frontal gyrus may serve as potential neuroimaging indicators for early identification of cognitive impairment in MHE patients, providing critical evidence for clinical screening protocols.

## Introduction

Minimal hepatic encephalopathy (MHE) is a neurological complication characterized by slow subclinical changes in extensive neurocognitive function in patients with end-stage liver cirrhosis. The disease has an insidious onset, initially presenting with mild psychomotor delay or inattention, characterized by reduced executive function and memory impairment (1). While not as immediately life-threatening as dominant hepatic encephalopathy, MHE significantly affects patients' health-related quality of life and daily functioning, potentially leading to driving errors and severe traffic accidents. In addition, previous research demonstrated that the incidence of MHE in patients with liver cirrhosis can reach 33%−50% (2). Without effective treatment, ~40% of patients progress to significant hepatic encephalopathy within 6 months (3, 4). When overt hepatic encephalopathy (OHE) develops, the 1-year mortality rate can reach 58% (5), and the duration of hospitalization is notably prolonged. Consequently, MHE serves as a predictor of higher risk for developing OHE, which is linked to poor prognosis and increased overall mortality. At present, MHE is the most overlooked detrimental subclinical form of hepatic encephalopathy. There is no gold standard for the diagnosis of MHE, and only neuropsychological test scores remain the least validated method, leading to imprecise results. Early and accurate identification, along with targeted intervention, is essential to reduce the likelihood of progression to OHE and to enhance patients' quality of life.

With advancements in magnetic resonance imaging (MRI), researchers have used resting-state functional MRI (rs-fMRI) to non-invasively examine changes in spontaneous brain activity in patients with brain dysfunction. Various algorithms, including amplitude of low-frequency fluctuation (ALFF), fractional ALFF (fALFF), percent amplitude of fluctuation (PerAF), and regional uniformity (ReHo), have been utilized to analyze rs-fMRI data. These methods offer valuable insights into the spontaneous activity of different brain regions (6, 7).

Over the past decade, an increasing number of rs-fMRI studies have examined changes in spontaneous brain activity in MHE patients with liver cirrhosis. However, inconsistencies have been observed across brain regions identified by different algorithms. For instance, Chen et al. (8) used ALFF analysis and found abnormal changes in the precuneus and adjacent cuneus, visual cortex, and left posterior cerebellum in MHE patients. Ni et al. (9) reported significantly lower ReHo values in the bilateral precuneus, supplementary motor areas, and precuneus of MHE patients using ReHo analysis. MHE patients exhibit widespread diffuse abnormalities in baseline brain activity across different brain regions (8–11). However, most of these differential brain regions lack specificity for the early detection of MHE. Even with the use of a fully quantitative neuroimaging meta-analysis that combines ALFF, fALFF, and ReHo to investigate changes in spontaneous brain activity, consistent findings across studies remain elusive. Cao et al. (12) conducted a meta-analysis involving ALFF, fALFF, and ReHo. They found that alterations in the fronto-striato-cerebellar and visual-sensorimotor networks could represent potential pathophysiological mechanisms of HE in cirrhotic patients. Qin et al. (13) only found that changes in spontaneous brain activity in the left superior frontal gyrus and median/paracingulate gyrus were associated with MHE. The non-specificity and inconsistencies in the identification of different brain regions hinder the development of a comprehensive understanding of the underlying neural mechanisms. Further research is necessary to progress in this field.

Furthermore, previous studies relied on univariate group-level difference analyses, which failed to capture the information embedded in the spatial distribution patterns of brain activity. Therefore, univariate differences between groups cannot replace the diagnostic assessment of MHE patients. This limitation contributes to the heterogeneity observed in research outcomes. In recent years, advances in machine learning methods have led to the widespread usage of algorithms that can thoroughly explore the potential value of data, playing a crucial role in clinical decision-making (14, 15). Common machine learning algorithms include support vector machines (SVM), K-nearest neighbor, naive Bayes, logistic regression, decision trees, and deep neural networks. Among them, SVM has demonstrated promising performance in assisting disease diagnosis, particularly when applied to specific disease types, characteristic attributes, and classification models (16). This approach incorporates multiple dimensions of brain data for cross-validation, enabling the identification of potential patterns in the dataset (17). It offers several advantages, including ease of implementation, robustness, superior classification performance, a low likelihood of overfitting, and excellent diagnostic accuracy, particularly in small to medium-sized datasets. These features make it an effective tool for analyzing and interpreting neuroimaging data. This makes SVM a valuable tool for studying neuroimaging biomarkers in the functional imaging of psychiatric disorders (18, 19). Therefore, machine learning-based classification of local brain activity characteristics in MHE patients, along with the integration of multi-dimensional spatial information, may provide more specific results for the early diagnosis of MHE. In the present study, MHE patients and healthy controls (HCs) were prospectively included. The sensitivity and specificity of spontaneous brain activity signals in rs-fMRI brain regions, including ALFF, fALFF, PerAF, and ReHo, were analyzed and compared for classification and diagnosis using machine learning methods. We hypothesized that PerAF would show superior diagnostic accuracy compared to other amplitude metrics in SVM-based classification. This study aimed to help clinicians in the early identification of MHE patients and to promote the development of clinical diagnostic and treatment techniques for MHE.

## Methods

### Study subjects

The study was approved by the Ethics Committee of the First Affiliated Hospital of Guangxi Medical University (Nanning, China), and it conforms to the provisions of the Declaration of Helsinki. All the study subjects provided written informed consent prior to enrollment. Patients were recruited from those hospitalized at the Second Affiliated Hospital of Guangxi Medical University between May 2022 and November 2023. A total of 48 cirrhotic patients with MHE and 42 HCs with no history of cirrhosis were enrolled. Only chronic cirrhotic patients without OHE were involved. The patients included in this study had hepatitis B-induced liver cirrhosis and did not take drugs that affect cognition. All subjects underwent rs-fMRI scans. Exclusion criteria are as follows: (a) a history of OHE (past or present, based on clinical assessment), (b) drug or alcohol abuse, (c) brain lesions, (d) known psychiatric disorders, and (e) head motion exceeding 3.0 mm or 3.0° during MRI scan.

MHE was diagnosed based on neuropsychological tests, including the Number Connection Test-A (NCT-A) and the Digit-Symbol Test (DST). The diagnosis of MHE was made when the scores on both tests were >2 standard deviations (SD), which was below the mean value of age-matched controls.

Three MHE patients were excluded from the study due to head motion (*n* = 1), data missing (*n* = 1), and poor image quality (*n* = 1). Furthermore, two HCs were excluded due to head motion (*n* = 1) and poor image quality (*n* = 1), leaving 45 MHE patients and 40 HCs in the final analysis.

### MRI data collection

MRI data were acquired using a 3 Tesla MR scanner (TIM Trio; Siemens Medical Solutions, Erlangen, Germany) equipped with a 32-channel head coil. All subjects were positioned in the standard head coil and secured with foam padding to minimize head movement. They were instructed to remain still, keep their eyes closed, and stay awake during the scan. High-resolution axial T1-weighted FLASH sequence images were obtained from each subject to detect clinically silent lesions. An interleaved ascending acquisition was used, with odd-numbered slices acquired first. Functional images were acquired using a gradient-echo echo-planar imaging (GRE-EPI) sequence sensitive to BOLD contrast (TR = 2,170 ms, TE = 30 ms, flip angle = 90°, FOV = 192 × 192 mm^2^, matrix = 64 × 64, slice thickness = 2 mm, slice gap = 0.3 mm). Axial scanning was performed over 70 layers, and 186 dynamic imaging scans were acquired.

### Data preprocessing

The rs-fMRI data processing was performed using the MATLAB (R2017b) platform (MathWorks, Natick, MA, USA; https://uk.mathworks.com/products/matlab). Data preprocessing and calculations were carried out using the RESTplus (v1.27) analysis toolkit (http://restfmri.net/forum/restplus) and Statistical Parametric Mapping (SPM 12, v7771, http://www.fil.ion.ucl.ac.uk/spm). The preprocessing steps included discarding the first 10 time points, slice timing correction, 24-parameter head-motion correction (includes rigid-body registration operations where the translation in the X, Y, and Z directions exceeds 3 mm and the rotation exceeds 3°, 12-parameter gradient non-linearity compensation modeling and 6-parameter B0 inhomogeneity correction). Then, spatial normalization to the standard Montreal Neurological Institute (MNI) template with a resampled voxel size of 3 × 3 × 3 mm3. Spatial smoothing was applied after calculating ReHo, although it was initially omitted. After completing the calculation, the ReHo image was smoothed, linear trends were removed, and nuisance covariates (including white matter signals, cerebrospinal fluid signals, the Friston-24 head motion parameters, and global mean signal) were regressed out (20). The data were thereafter filtered using the conventional frequency band (0.01–0.08 Hz) (19, 21). The preprocessed fMRI data was further applied to the next analysis.

### Data calculations

After preprocessing, ALFF, fALFF, PerAF, and ReHo analyses were computed using RESTplus software. We obtained the ALFF value as follows: First, convert the time series of whole brain signal strength into the frequency domain power spectrum through a fast Fourier transform (FFT). The power spectrum was calculated, and the mean square root was used as the ALFF value. However, the fALFF value of each voxel was obtained by dividing the ALFF value under 0.01–0.08 Hz frequency spectrum by that of the entire frequency band. Finally, zALFF and zfALFF, known as standardized values, were obtained by subtracting the global mean value and further dividing it by the standard deviation.

PerAF value refers to the percentage of resting-state BOLD fluctuation about the mean signal intensity over the entire time series. We calculated the PerAF of each voxel with the following equations. The PerAF of each voxel was calculated as follows:


(1) $$\text{PerAF}=\left(\frac{1}{N}\sum_{i=1}^{N}\left|\frac{X_{i}-\mu }{\mu }\right|\right)\times 100\%$$

(2) $$\begin{array}{lll} \mu & = & \frac{1}{N}\sum_{i=1}^{N} \end{array}$$

Note: *X*_*i*_ = signal intensity at the time point *i*, μ = mean value of the time series, *N* = total number of time points of the time series.

ReHo values across the whole brain were calculated using Kendall's coefficient of concordance at a voxel-wise level in three frequency bands to assess the similarity of the time series of its 26 nearest voxels (27 voxels are more sensitive than 19 and 7 voxels) (22). The standardized ReHo value was obtained by dividing each subject's ReHo value by the mean value of the entire brain. Finally, the ReHo maps for each participant were smoothed by a Gaussian filter of 6-mm full width at half maximum (FWHM) to reduce noise and residual differences and were used in statistical analysis (23).

### Statistical analysis

#### Demographic variables

The statistical analysis was performed using SPSS 26.0 software (IBM, Armonk, NY, USA). Categorical variables were presented as count (*n*), while continuous variables were expressed as the mean ± SD. The Chi-square test was employed to compare the distribution of genders between the MHE and HC groups. A two-sample *t*-test was utilized to compare differences in age, years of education, and neuropsychological scores between the two groups. All tests were two-tailed, and *P* < 0.05 was considered statistically significant.

Two-sample *t*-tests were performed to detect the differences of the ALFF, fALFF, PerAF, and ReHo maps between MHE and HCs using RESTplus software. Frame-wise displacement (FD, Jenkinson) parameters were regressed to avoid the influence of head motion, and the ALFF, fALFF, PerAF, and ReHo results were corrected for multiple comparisons by the Gaussian Random Field (GRF) (voxel *P* < 0.01, cluster *P* < 0.01, cluster size > 100 voxels, two tailed).

#### Classification analysis using SVM

As a supervised learning method designed for class separation, SVM differs from traditional statistical approaches by mapping non-linear data into a high-dimensional feature space and identifying the optimal hyperplane with maximum marginal separation for effective data classification. The goal of hyperplane localization is to get the support vector as far away from the nearest data point as possible. Nested cross-validation was used in this study. The outer loop is used for the final classification performance evaluation, the inner loop is used for feature selection and model parameter tuning, and the intergroup comparison (*t*-test) and ROI screening are only performed within the training fold of the outer loop. The leave-one-out method was used to split the training set and test set data; the grid search method and cross-validation method were used to find the optimal parameters *c* (penalty coefficient) and *g* (γ). In this process, the inner loop performs the cross-validation of the best hyperparameter function and provides the best hyperparameters of the model to the outer loop. After multiple cross-validations of the outer loop, the optimal hyperparameters of the model can be obtained, which can not only prevent data information leakage but also obtain relatively low model deviation. Therefore, our model still has good stability and reliability on new and independent data sets. The LIBSVM package (libsvm 3.24, https://www.csie.ntu.edu.tw/~cjlin/libsvm/) was used on the MATLAB 2017b platform to develop the classifier. The area under the receiver operating curve (AUC) was evaluated to test the predictive performance of the established model.

## Results

### Demographic and clinical characteristics

Demographic and clinical data are summarized in Table 1. No significant differences were observed in age (*P* = 0.395), gender (*P* = 0.577), and education year (*P* = 0.732) between the two groups. The NCT-A and DST scores exhibited significant differences between the two groups (both *P* < 0.001).

**Table 1:** Demographic and clinical data of cirrhotic patients and healthy controls.

**Variables**	**MHE (*n =* 45)**	**HCs (*n =* 40)**	***P*-value**
Gender (male/female)	42/3	36/4	0.577^a^
Age (years)	52.76 ± 8.49	50.25 ± 7.57	0.395^b^
Education (years)	11.47 ± 2.78	11.85 ± 2.91	0.732^b^
NCT-A (score)	64.00 ± 13.37	35.95 ± 10.98	< 0.001^b^
DST (score)	24.78 ± 4.13	39.13 ± 5.15	< 0.001^b^
Child-Pugh (score)	8.00 ± 1.93	–	–

^a^The *P*-value of the two groups was calculated by the Chi-square test.

^b^The *P*-value of the two groups was calculated by a two-sample t-test.

MHE, minimal hepatic encephalopathy; HC, healthy control; DST, digital symbol test; NCT-A, number connection test-A.

### Abnormal regional brains

**Figure 1:**
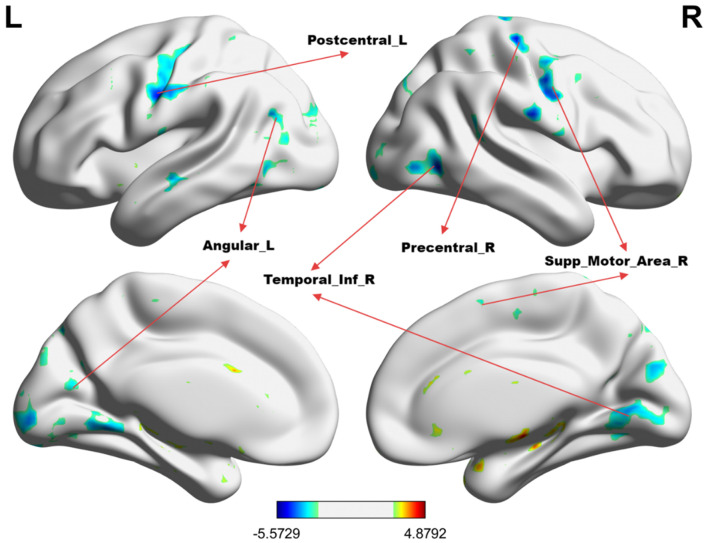
The group differences in ALFF value between MHE patients and HCs. **Cold colors** indicate decreased ALFF in the left angular gyrus, right inferior temporal gyrus, left postcentral gyrus, left precentral gyrus, and right supplementary motor area (voxel *P* < 0.01, cluster *P* < 0.01, GRF correction, cluster size > 100 voxels).

**Figure 2:**
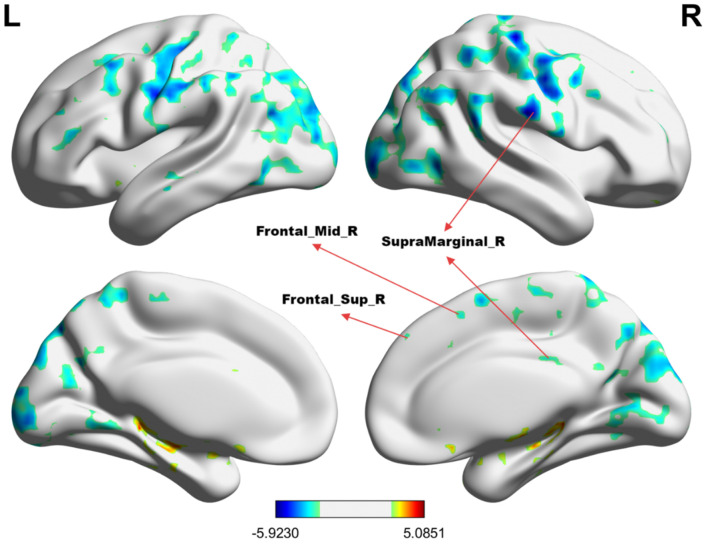
The group differences in PerAF value between MHE patients and HCs. **Cold colors** indicate decreased PerAF in the right supramarginal gyrus, right dorsolateral superior frontal gyrus, and right middle frontal gyrus (voxel *P* < 0.01, cluster *P* < 0.01, GRF correction, cluster size > 100 voxels).

**Figure 3:**
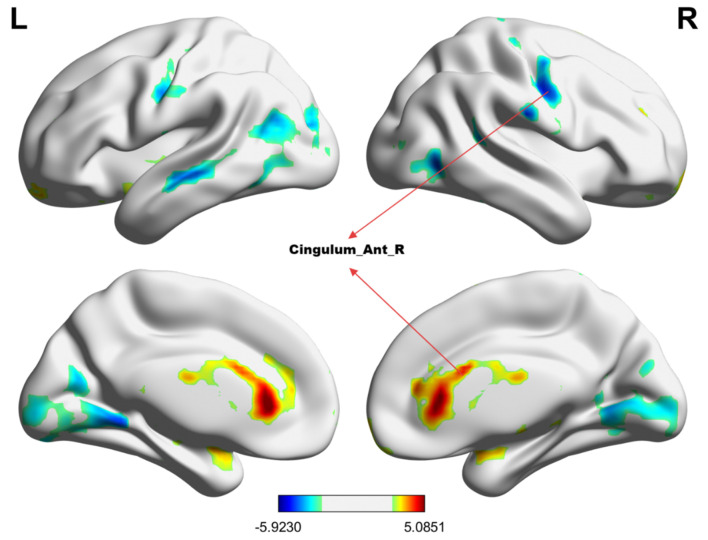
The group differences in ReHo value between MHE patients and HCs. **Cold colors** indicate decreased ReHo in the right anterior cingulate and paracingulate gyrus (voxel *P* < 0.01, cluster *P* < 0.01, GRF correction, cluster size > 100 voxels).

Table 2 summarizes the altered regions in the whole-brain analysis applying the four-amplitude metrics. Compared to the HCs group, MHE patients showed decreased ALFF in the left angular gyrus, right inferior temporal gyrus, left postcentral gyrus, precentral gyrus, and right supplementary motor area; decreased PerAF in the right supramarginal gyrus, right dorsolateral superior frontal gyrus, and right middle frontal gyrus; decreased ReHo in the right anterior cingulate and paracingulate gyrus. There were no brain regions with significant differences in the fALFF metrics between the two groups. The details were presented in Figures 1–3.

**Table 2:** Abnormal brain regions in the MHE patients compared to HCs.

	**Regions**	**MNI co-ordinate (mm) (X, Y, Z)**	**Cluster size**	**Peak *t*-value**
ALFF	Angular_L	(−48, −69, 24)	215	−4.187
	Temporal_Inf_R	(48, −66, −3)	122	−5.000
	Postcentral_L	(−45, −9, 33)	336	−5.573
	Precentral_R	(33, −24, 63)	278	−4.988
	Supp_Motor_Area_R	(3, 6, 60)	321	−5.564
fALFF	–	–	–	–
PerAF	SupraMarginal_R	(60, −18, 24)	9921	−5.923
	Frontal_Sup_R	(27, 66, 12)	306	−4.865
	Frontal_Mid_R	(36, 39, 42)	106	−4.775
ReHo	Cingulum_Ant_R	(6, 15, 24)	11212	5.712

Differences between the MHE and HCs group were assessed for significance using the two-sample t-test, and the statistical threshold was corrected for multiple comparisons with GRF (voxel *P* < 0.01, cluster *P* < 0.01, cluster size > 100 voxels).

AAL, Automated anatomical labeling; MNI: Montreal Neurological Institute; ALFF: Amplitude of low-frequency fluctuation; fALFF: Fractional amplitude of low-frequency fluctuation; PerAF: Percent amplitude of fluctuation; ReHo: Regional homogeneity. Angular_L: left angular gyrus; Temporal_Inf_R: right inferior temporal gyrus; Postcentral_L: left postcentral gyrus; Precentral_R: right precentral gyrus; Supp_Motor_Area_R: right supplementary motor area; SupraMarginal_R: right supramarginal gyrus; Frontal_Sup_R: right dorsolateral superior frontal gyrus; Frontal_Mid_R: right middle frontal gyrus; Cingulum_Ant_R: right anterior cingulate and paracingulate gyrus.

### SVM analysis

**Figure 4:**
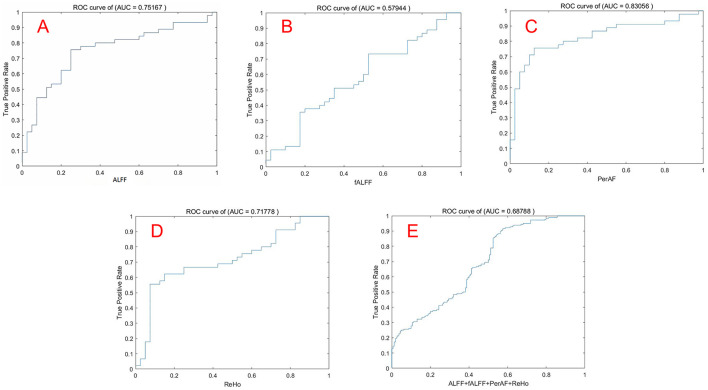
The ROC curve was used to evaluate the performance of the four indicators by SVM. Note: **(A)** ALFF; **(B)** fALFF; **(C)** PerAF; **(D)** ReHo; **(E)** Combination of ALFF, fALFF, PerAF, ReHo. ROC, Receiver operating characteristic; SVM, Support vector machine.

The mean ALFF, fALFF, PerAF, and ReHo values of altered brain regions were used as features separately or together in the SVM models distinguishing the MHE patients and HCs. Table 3 and Figure 4 illustrate the results of the SVM classification between 45 MHE patients and 40 HCs. Thus, the best discrimination was obtained when PerAF metrics (Figure 4C) in the right supramarginal gyrus, right dorsolateral superior frontal gyrus, and right middle frontal gyrus were used as features for an accuracy of 81.18%, a sensitivity of 75.56% and a specificity of 87.50% respectively. This was followed by the classification based on ALFF and fALFF (Figures 4A, B), which achieved an accuracy of 75.29% and 60.00%, sensitivity of 75.56% and 73.33%, and specificity of 75.00% and 45%. For the ReHo measures (Figure 4D), the accuracy, sensitivity, and specificity were 70.59%, 66.67%, and 75.00%, respectively. In addition, the SVM results showed that all the combinations exhibited an accuracy of 68.53%, sensitivity of 91.67%, and specificity of 42.50% (Figure 4E).

**Table 3:** SVM classification performance across the four different amplitude metrics, single and combined.

**Metrics**	**AUC**	**Accuracy**	**Sensitivity**	**Specificity**	**Precision**
ALFF	0.75	75.29%	75.56%	75.00%	77.27%
fALFF	0.58	60.00%	73.33%	45.00%	60.00%
PerAF	0.83	81.18%	75.56%	87.50%	87.18%
ReHo	0.72	70.59%	66.67%	75.00%	75.00%
Total	0.69	68.53%	91.67%	42.50%	64.20%

Total represents ALFF+fALFF+PerAF+ReHo.

## Discussion

In this study, four regional amplitude metrics, including ALFF, fALFF, PerAF, and ReHo, were used to examine the spontaneous brain functional activities of MHE patients and explore their potential diagnostic value with a machine learning approach. Our findings revealed a significant decrease in regional brain activity in MHE patients compared to HCs. Notably, the PerAF metric demonstrated the highest diagnostic accuracy among the four metrics, achieving an accuracy of 81.18%, sensitivity of 75.56%, and specificity of 87.50% when applied to the right supramarginal gyrus, right dorsolateral superior frontal gyrus, and right middle frontal gyrus. These results provide novel insights into the neural mechanisms underlying MHE and highlight the PerAF metrics as imaging biomarkers for distinguishing MHE patients from HCs. Our samples provide case data for the development of AI-assisted diagnostic systems, enabling the automatic labeling of abnormal PerAF brain regions in the future.

### Diagnostic value of local brain activity metrics

Notably, fALFF reflects the ratio of low-frequency amplitude to the total amplitude across the full frequency band (24), selectively reducing interference from non-specific signals, such as those from ventricles, which enhances sensitivity and specificity in detecting spontaneous brain activity. However, the classification accuracy of fALFF in this study was generally lower than that of other localized measures, which could be attributed to the influence of full-band power fluctuations on fALFF calculations and residual effects of cardiac and respiratory cycles, obscuring accurate amplitude characteristics for a given frequency band. The lack of significant differences in fALFF between MHE patients and HCs suggests that this metric may be less sensitive to the neural alterations associated with MHE. This finding highlights the importance of selecting appropriate amplitude metrics for studying specific neurological conditions. Moreover, PerAF represents the percentage of Blood Oxygen Level-Dependent (BOLD) signal fluctuation relative to the average BOLD signal strength over a specific time series and, based on ALFF, reflects the extent of temporal variation in BOLD signal in individual voxels. As a recently developed analytical method known for its effectiveness, stability, and reliability, our findings demonstrate that PerAF, a novel metric for measuring the periodicity of spontaneous brain activity, shows increased sensitivity in detecting subtle neural changes associated with MHE. The present results are consistent with the test-retest reliability findings reported by Jia et al., further confirming the robustness of this methodological approach (25) and indicating that the PerAF algorithm outperformed both fALFF and ALFF, demonstrating the greatest efficacy in tracking changes in local brain activity in MHE patients. Interestingly, the combination of ALFF, fALFF, PerAF, and ReHo metrics did not significantly improve diagnostic accuracy compared to PerAF alone. This may be due to the overlapping information captured by ALFF and fALFF, which both measure the amplitude of low-frequency fluctuations in the BOLD signal. In contrast, PerAF focuses on the periodicity of these fluctuations and directly quantifies the absolute percentage fluctuation of the BOLD signal amplitude. This approach can more sensitively capture the transient hemodynamic response triggered by neural activity and is particularly effective at detecting temporal signal variations related to neurovascular coupling. ALFF, fALFF, PerAF, and ReHo are frequently utilized algorithms in rs-fMRI analysis, each providing complementary insights into the spontaneous activity of specific brain regions (6). Given their analogous quantitative interpretations in brain physiology, using machine learning to identify indicators with high statistical classification efficiency, as well as combining them to explore intrinsic brain activities, offers significant promise.

### Altered brain regions and significance

The observed alterations in brain activity in MHE patients align with the known cognitive and motor deficits associated with the condition. The left angular gyrus is involved in higher-order cognitive functions such as language processing, attention, and memory (26). Similarly, the right inferior temporal gyrus, which plays a role in visual processing and object recognition, showed reduced ALFF, potentially contributing to the visuospatial deficits often observed in MHE patients (27). Zafiris et al. (28) used task-state functional magnetic resonance imaging and identified disrupted interactions involving the right inferior parietal cortex with the parietal-occipital cortex, medial parietal sulcus, anterior cingulate cortex, right prefrontal cortex, medial temporal lobe, and V5 of the extrastriate cortex in cirrhotic patients without OHE, providing a theoretical basis for the early visual deficits found in these patients. The decreased ALFF observed in this region in MHE patients may reflect impaired cognitive integration and information processing.

The left postcentral gyrus and left precentral gyrus, which are primary somatosensory and motor cortices, respectively, also exhibited decreased ALFF. Both are crucial for sensory-motor integration, helping us in perceiving the environment and executing appropriate motor responses. These findings suggest that MHE may disrupt sensory and motor processing, consistent with previous reports of motor coordination deficits in MHE patients (29). Additionally, the right supplementary motor area (SMA), which is involved in motor planning and execution, showed decreased PerAF. This abnormality may underlie the motor dysfunction and psychomotor slowing frequently observed in MHE (30).

The right dorsolateral superior frontal gyrus and right middle frontal gyrus, both parts of the prefrontal cortex, are critical for executive functions such as working memory, decision-making, and cognitive control (31). Similarly, the reduced PerAF in the prefrontal cortex and right supplementary motor area may indicate impaired top-down control and motor planning, contributing to the executive and motor deficits in MHE. The decreased PerAF in these regions may explain the executive dysfunction commonly seen in MHE patients. Furthermore, as an important node in the executive network, the anterior cingulate cortex (ACC) is involved in the core area of attention, related to attention control, response inhibition, error detection, and emotional processing (32). Moreover, psychomotor speed and attention deficits are a major manifestation of patients with mild HE (33). The right anterior cingulate and paracingulate gyrus showed reduced ReHo, potentially contributing to the emotional and behavioral disturbances in MHE. Multiple investigators using positron emission computed tomography (PET), fMRI, and magnetic resonance spectroscopy (MRS) have found that ACC dysfunction in cirrhotic patients with or without HE may be one of the causes of neurocognitive dysfunction and may be associated with metabolic disorders (32–35). Interestingly, we found that there seemed to be some brain network connectivity patterns in these abnormal brain regions. Chen et al. (36, 37) reported that MHE patients had a significant decrease in functional connectivity of the default mode network (DMN), and this decrease became more severe with the development of the disease. At the same time, MHE patients not only show reduced functional connectivity of the DMN, but also participate in the disorder of the cognitive control network, sensory motor network (SMN), and subcortical network (38, 39). Combined with the results of previous research, our study may suggest that the functional connectivity of the brain network in MHE patients is abnormal compared with healthy people. In particular, functional decline in the left angular gyrus, right inferior temporal gyrus, right precentral gyrus, right supplementary motor area, right superior sellar gyrus, and right middle frontal gyrus may reflect disruption of large-scale brain networks critical for the maintenance of cognitive function, such as the SMN, DMN, dorsal attention network (DAN), and visual attention network (VAN). These abnormalities may be closely related to the cognitive dysfunction of patients, and further research is necessary to confirm this.

## Limitations of the study

This study has certain limitations. First, the sample size was relatively limited; we only used internal validation data to make the test set when applying an SVM method, and external validation data might be more reliable and robust. Second, the patients included in this study were end-stage cirrhosis patients, and the etiology was not classified, which may lead to a bias in the classification results, because the brain damage caused by liver cirrhosis with different etiologies can also be slightly different. Third, we only used regional brain activity metrics to identify MHE patients; the applicability of the proposed approach to different subtypes of hepatic encephalopathy remained elusive. Changes of brain intrinsic networks and the multimodal imaging technique with structural and functional coupling neuroimaging datasets may further improve classification. Future studies can be conducted based on the above limitations.

## Conclusions

This study used machine learning techniques to investigate the diagnostic classification of spontaneous regional brain activity alterations in patients with MHE. The observed reduction in the PerAF within the right supramarginal gyrus, right dorsolateral superior frontal gyrus, and right middle frontal gyrus demonstrated superior diagnostic accuracy, underscoring its potential utility as a reliable biomarker for MHE. These findings enhance our understanding of the neural mechanisms underlying MHE. Future research will focus on multimodal brain function monitoring (DTI+rs-fMRI) combined with machine learning methods.

## Data Availability

The original contributions presented in the study are included in the article/supplementary material, further inquiries can be directed to the corresponding author.
